# Postoperative Imaging of Bone and Soft Tissue Tumors in the Extremity: A Comprehensive Review

**DOI:** 10.3390/diagnostics14242794

**Published:** 2024-12-12

**Authors:** Seul Ki Lee, Min Wook Joo, Jee-Young Kim, Nicholas Matthew Bernthal

**Affiliations:** 1 Department of Radiology, St. Vincent’s Hospital, College of Medicine, The Catholic University of Korea, 222 Banpo-daero, Seocho-gu, Seoul 06591, Republic of Korea; 2 Department of Orthopaedic Surgery, St. Vincent’s Hospital, College of Medicine, The Catholic University of Korea, 222 Banpo-daero, Seocho-gu, Seoul 06591, Republic of Korea; 3 Department of Orthopaedic Surgery, University of California Los Angeles, Los Angeles, CA 90095, USA

**Keywords:** bone tumor, soft tissue tumor, musculoskeletal tumor, postoperative imaging, limb salvage, endoprosthesis, complications, local recurrence

## Abstract

Postoperative imaging of musculoskeletal tumors poses a significant diagnostic challenge for radiologists. The complexity arises from the need to differentiate between expected postoperative changes, potential complications, and local recurrence. The choice of imaging modality depends on the type of primary tumor. Standard radiological modalities such as radiography, computed tomography (CT), and magnetic resonance imaging (MRI) are widely utilized. Radiography and CT are especially valuable for assessing primary bone tumors, as they provide detailed views of bone structures and alignment, as well as revealing postoperative complications. MRIs are particularly effective for evaluating soft tissue tumors and identifying local recurrences due to its superior soft tissue contrast. The advanced imaging techniques, such as diffusion-weighted imaging and dynamic contrast-enhanced MRI, have significantly improved diagnostic accuracy in detecting tumor recurrence. An in-depth understanding of surgery-specific imaging findings, as well as the ability to detect recurrent disease, is crucial for early diagnosis of complications and improved patient outcomes. Familiarity with normal postoperative changes helps radiologists distinguish them from abnormal findings indicative of complications or tumor recurrence. This review article aims to outline the surgical options for musculoskeletal tumors, detail the various imaging techniques used in postoperative surveillance, and discuss the potential complications. By understanding the role of different imaging modalities and their applications associated with various surgical procedures, clinicians and radiologists can provide accurate and timely diagnoses.

## 1. Introduction

Postoperative imaging of bone and soft tissue tumors presents a complex diagnostic challenge for radiologists, necessitating a comprehensive understanding of various imaging modalities and their applications [1]. The choice of imaging modality during the postoperative period depends on the specific tumor type [2]. While plain radiography, computed tomography (CT), and magnetic resonance imaging (MRI) are commonly used, their relative importance varies [3]. Plain radiography and CT are particularly valuable for monitoring bone tumors as they can effectively assess bone structures and detect changes that may indicate complications or recurrence [4]. MRIs, on the other hand, are often the preferred modality for soft tissue tumor follow-up due to its superior ability to differentiate between soft tissue structures and detect subtle postoperative changes [3]. Early detection of recurrence is crucial for optimal patient management [5], and a systemic approach that includes a thorough review of the patient’s clinical and surgical history, as well as preoperative imaging studies, is essential [6]. This background information helps radiologists differentiate between local recurrences and postoperative changes, which can often mimic different conditions [7].

This review article aims to provide radiologists with a structured approach to imaging interpretation in patients who have undergone surgical treatment for bone and soft tissue tumors. By understanding the various postoperative changes and the subtle signs of recurrence, radiologists can contribute to improving patient outcomes.

## 2. Surgical Procedures According to the Biological Behavior of the Tumor

**Figure 1:**
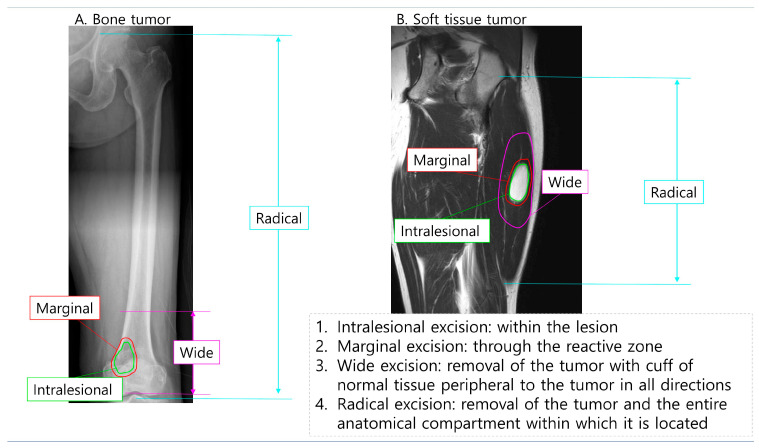
The four fundamental types of excisions in musculoskeletal tumor surgery. ((**A**): Bone tumor, (**B**): soft tissue tumor). The lines indicate the dissection plane and the volume of tissue removed in each procedure.

There are four fundamental types of excisions in musculoskeletal tumor surgery [8], each classified based on the relationship between the surgical plane and the tumor, including its pseudocapsule. These types differ in the amount of normal tissue removed and the potential for residual disease (Figure 1).

1. Intralesional excision: This procedure involves removing only a portion of the tumor, leaving behind the pseudocapsule and macroscopic tumor tissue. It is typically used for diagnostic purposes or for the subtotal removal of a tumor.
2. Marginal excision: In this procedure, the dissection plane passes through the pseudocapsule of the tumor, meaning the surgeon removes the tumor at its gross edge but may leave behind microscopic disease. This approach is suitable for general benign tumors or locally invasive benign tumors, such as the giant cell tumor of bone (GCTB), especially when combined with adjuvant therapy. However, it is not appropriate for malignant tumors due to the risk of residual tissue.
3. Wide excision: This is the preferred approach for malignant tumors. It involves removing the entire tumor, its pseudocapsule, and a surrounding margin of normal tissue in all directions to minimize the risk of leaving behind tumor cells. The ideal width of the margin is debated among experts, as it can depend on the type and location of the tumor. The goal is to achieve clear margins while balancing the extent of normal tissue resected with the need to preserve function.
4. Radical excision: This more extensive procedure entails removing the tumor and the entire anatomical compartment in which it is located. For example, if a tumor is in a muscle compartment, the entire muscle group may be excised. Radical excision is typically reserved for aggressive or advanced tumors where complete anatomical clearance is necessary.

In general, benign tumors can be effectively treated with intralesional excision or marginal excision, particularly if they are not expected to recur or invade surrounding tissues [8]. However, for locally invasive benign tumors, such as GCTB, a marginal excision may be performed, sometimes combined with additional adjuvant therapies like cryotherapy or chemical adjuvants to reduce the risk of recurrence [9].

For malignant tumors, the standard approach is a wide excision to ensure that the tumors, their pseudocapsule, and a margin of surrounding tissue are completely removed [10]. The goal is to prevent local recurrence and maximize the chances of a cure. Importantly, wide excisions can be performed as a part of a limb-salvage procedure, in which surgeons aim to preserve as much of the limb’s function and structure as possible. However, in some cases, amputation may be considered to achieve clear margins, although it does not necessarily offer a survival benefit over a limb-salvage procedure [11].

In situations where a wide excision is not feasible due to the tumor’s location or the potential damage to critical structures, such as nerves, blood vessels, or major joints, surgeons may intentionally leave behind marginal areas of the tumor. This is followed by adjuvant therapies, such as chemotherapy or radiation, to eradicate any residual tumor cells. Staging studies play a vital role in determining the extent of the tumor and the surrounding anatomy. These studies guide the choice of surgical procedure to achieve optimal margins while preserving as much normal tissue and function as possible [8,12].

## 3. Expected Postoperative Imaging Findings

### 3.1. Bone Tumors

For benign bone tumors, the Enneking classification system divides them into three stages based on their aggressiveness and potential for recurrence [8]. Stage 1 lesions are classified as latent and are often discovered incidentally during imaging for unrelated issues. A classic example of a Stage 1 lesion is the fibrous cortical defect. These lesions present as well-defined, geographic, lytic lesions with a surrounding sclerotic rim on radiographs. Stage 2 lesions are characterized by active growth and include conditions such as the aneurysmal bone cyst (ABC) and some grades of GCTBs. Radiographically, these lesions also appear as well-defined (geographic) but tend to expand the bone without a sclerotic rim. Stage 3 lesions are more aggressive and include high-grade GCTBs and aggressive ABCs. These lesions exhibit more extensive bone destruction, often with cortical disruption, and tend to have a higher recurrence rate after treatment. Bone-RADS is a reporting system used by radiologists to categorize and assess bone tumors on imaging studies, helping to determine the likelihood of malignancy and aid in diagnosis and treatment [13].

**Figure 2:**
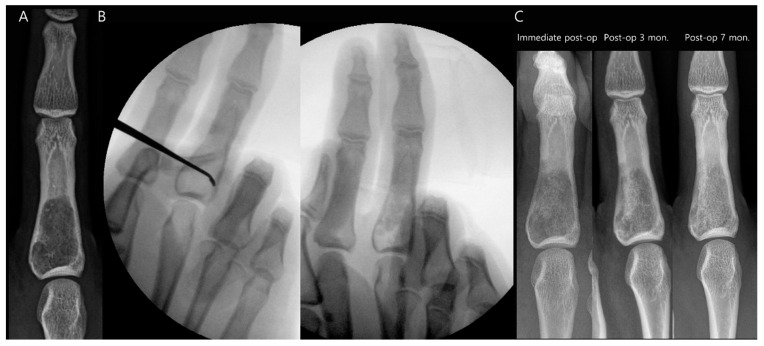
Intralesional curettage for enchondroma of the proximal phalanx of the middle finger. (**A**) Preoperative plain radiograph shows a well-defined osteolytic lesion containing chondroid calcification within the medullary cavity. (**B**) Intraoperative fluoroscopy shows complete curettage of the lesion, followed by autograft insertion. (**C**) Postoperative serial plain radiographs demonstrate successful incorporation of the graft 7 months after surgery.

**Figure 3:**
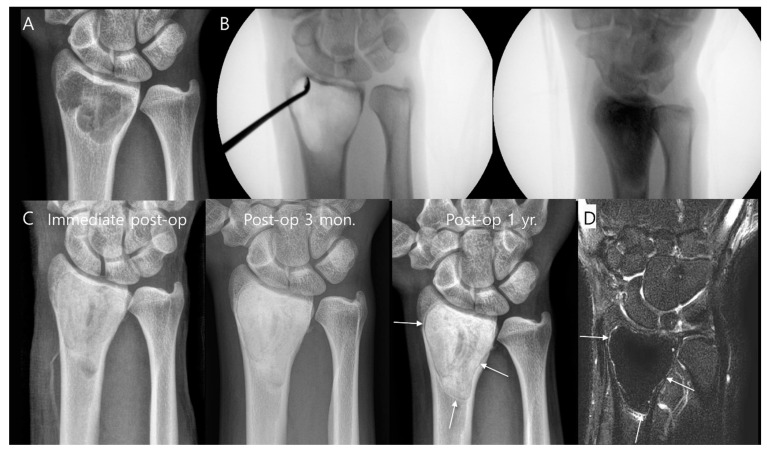
Intralesional curettage for giant cell tumor of the distal radius. (**A**) Preoperative plain radiograph shows a well-defined osteolytic lesion located in the epi-metaphysis of the distal radius. (**B**) Intraoperative fluoroscopy shows extended curettage with adjuvant treatment and cementation. (**C**) Postoperative serial plain radiographs demonstrate a thin, radiolucent line around the cement implant 1 year after surgery (arrows). (**D**) Postoperative coronal T2-weighted fat-suppressed MR image also shows a thin, hyperintense rim around the cement implant (arrows), referred to as a “clear zone”.

The most common surgical procedure for benign bone tumors is intralesional curettage-resection, followed by filling the bone defect with a bone graft [14]. Bone graft procedures can be performed using either autogenous bone grafts (harvested from the patient) or allografts (obtained from a donor and sterilized for medical use) [15]. For benign tumors with a very low risk of recurrence, such as nonossifying fibromas or unicameral bone cysts, simple curettage followed by bone grafting is often sufficient if surgical treatment is considered [16]. The majority of patients exhibited successful incorporation of the graft with the host bone after 6 months of surgery (Figure 2) [17]. However, more aggressive benign bone tumors, including ABCs and GCTBs, often require extended curettage with adjunctive therapies to reduce the risk of recurrence. These adjunctive therapies may include the use of polymethylmethacrylate cement, phenol, liquid nitrogen, and an argon beam coagulator [16]. Patients who undergo cement reconstruction may exhibit a radiolucent zone at the interface between the bone and the cement implant. This zone, which can measure up to 2 mm in width without progression, is considered a normal finding and represents a fibrovascular granulation wall. It does not indicate loosening of the implant and is often referred to as a “clear zone” (Figure 3) [18,19]. In some cases, internal fixation may be necessary to provide additional stability to the affected bone [14].

For malignant bone tumors in the extremities, amputation was the standard surgical approach in the past [20]. However, advancements in medical imaging and surgical techniques have enabled more precise oncologic resections of malignant bone tumors, leading to the widespread adoption of limb-salvage surgery [21]. Limb-salvage surgery refers to various surgical techniques aimed at removing a tumor with adequate margins and subsequently reconstructing the affected limb to preserve oncologic integrity, functionality, and a cosmetically acceptable appearance [22]. After the tumor has been resected, the surgeon must reconstruct the resulting surgical defect to restore the limb’s function.

**Figure 4:**
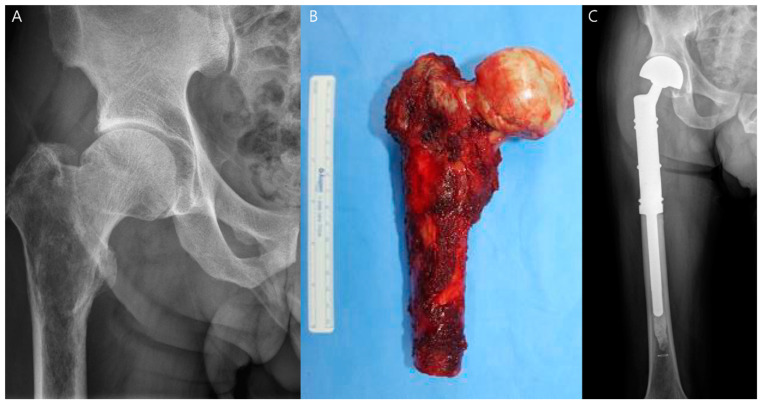
Tumor prosthesis at the proximal femur due to osteosarcoma of the proximal femur. (**A**) Preoperative plain radiograph shows an osseous tumor in the proximal femur, highly suggestive of osteosarcoma. (**B**) Gross clinical photograph of the widely excised tumor is shown. (**C**) Postoperative plain radiograph after endoprosthetic reconstruction of the proximal femur is shown.

**Figure 5:**
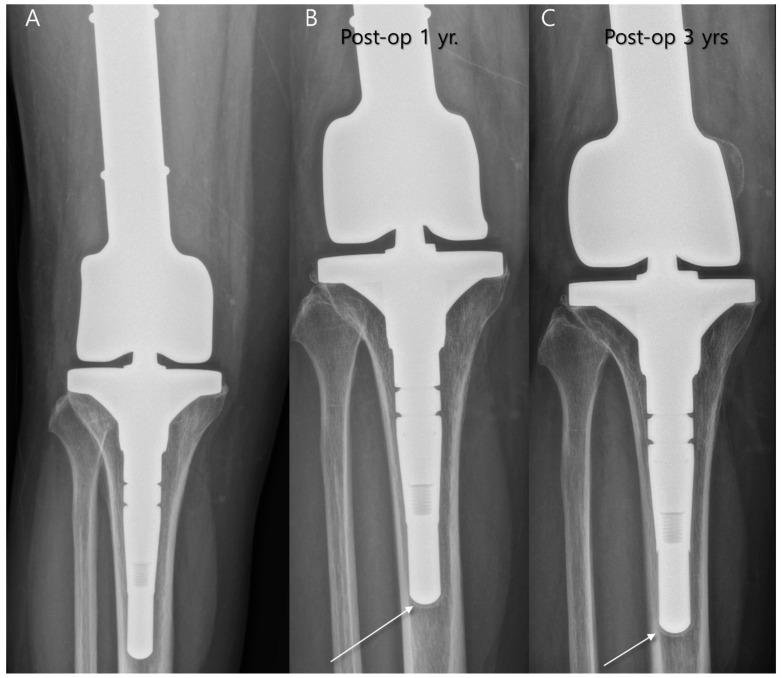
Endoprosthetic replacement at the distal femur due to malignancy in giant cell tumor. (**A**) Immediate postoperative plain radiograph is shown. (**B**) One-year follow-up plain radiograph shows a thin radiolucent zone at the metal–bone interface, measuring less than 2 mm (arrow). (**C**) Three-year follow-up plain radiograph shows no progression (arrow), indicating fibrous tissue ingrowth rather than implant loosening.

Replacing the excised bone segment requires consideration of optimal functionality. For smaller bone segments, reconstruction may be achieved with bone grafts, whereas larger defects often require the use of endoprostheses [20]. Endoprostheses are substantial metallic implants designed to replace the excised bone length, including the adjacent joint structure, supporting a more complete anatomical and functional restoration (Figure 4) [23]. Today, modular, off-the-shelf implants are widely available, although custom-made implants remain necessary in certain cases, especially when a growing prosthesis is needed to accommodate pediatric patients’ developmental needs [24]. At the interface between the implant and the bone, there is often a growth of fibrous tissue, which is the body’s natural response to foreign objects. This fibrous tissue layer can reach a thickness of up to 2 mm, which is generally considered acceptable and does not significantly compromise the long-term success and stability of the implant (Figure 5) [25].

### 3.2. Soft Tissue Tumors

ST-RADS is a reporting system used by radiologists to categorize and assess soft tissue tumors on imaging studies, helping to determine the likelihood of malignancy and guide subsequent treatment [26]. For many benign soft tissue tumors, watchful observation without immediate intervention is often sufficient. However, when surgery is necessary, most benign soft tissue tumors are removed through a marginal excision. This procedure involves the en bloc removal of the tumor along with the surrounding reactive zone, which is a thin capsule around tumors like lipomas or areas of altered signal around a mass on MRI scans. This approach is commonly applied to benign soft tissue tumors, including neurofibromas and lipomas [27]. In cases of more aggressive, intermediate soft tissue tumors, such as desmoid-type fibromatosis, wide excision is typically preferred. This technique involves not only removing the tumor but also a margin of normal tissue around it, ensuring more extensive removal to minimize the risk of recurrence [28]. The preferred approach for treating malignant soft tissue tumors in the extremity is a wide excision, ideally through limb-salvage surgery, often accompanied by radiation therapy [29]. A key component of effective limb-salvage surgery is successful reconstruction, as this allows for the removal of large tumors or tumors involving critical structures without compromising the limb’s functionality [30].

**Figure 6:**
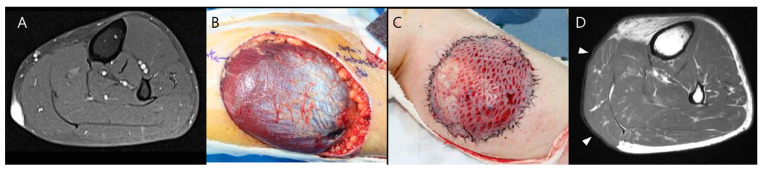
Wide excision for dermatofibrosarcoma protuberans on the calf area. (**A**) Preoperative axial T1-weighted enhanced MR image shows a well-enhancing soft tissue tumor confined to the skin and subcutaneous fat layer. (**B**) Tumor bed excision was performed due to the patient’s previous unplanned excision at another hospital, where initial management was undertaken. (**C**) To address the resultant defect, a split-thickness skin graft was applied to ensure adequate coverage. (**D**) Postoperative axial T1-weighted MR image after 1 year shows successful skin coverage at the operative site (arrowheads).

**Figure 7:**
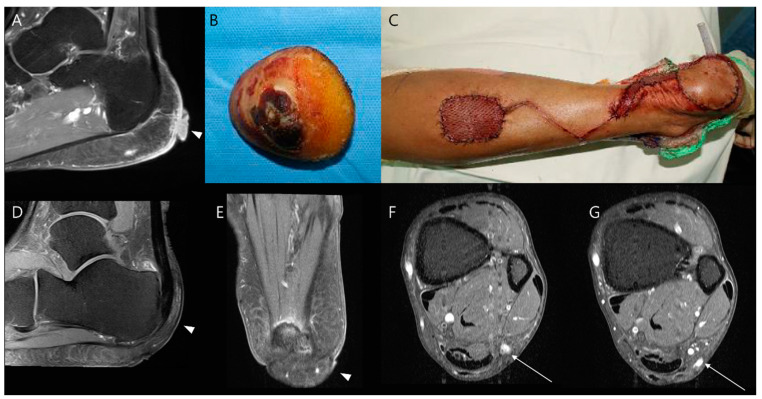
Wide excision for malignant melanoma on the heel. (**A**) Preoperative sagittal T1-weighted enhanced MR image shows a fungating mass arising from the skin (arrowhead) with infiltration into the subcutaneous fat layer. (**B**,**C**) The tumor was removed through wide excision, and a pedicled flap (reverse sural flap) was used to cover the resultant defect. (**D**,**E**) Postoperative sagittal and axial T1-weighted enhanced MR images illustrate successful coverage of the defect by the flap (arrowheads), confirming proper flap integration. (**F**,**G**) Axial T1-weighted enhanced MR images in cranial and caudal views further verify that blood vessels originating from the sural artery (arrow) are appropriately connected to the flap area (arrow).

Several reconstructive techniques have evolved over time [31,32,33,34,35]. For small or superficial defects, primary closure or split-thickness skin grafts are commonly used (Figure 6) [36]. Larger defects, resulting from more extensive resections, often require myocutaneous flaps [37]. Myocutaneous flaps are particularly advantageous because they provide robust coverage for extended resections, as well as the structural integrity needed for long-term outcomes. Pedicled flaps are beneficial for preserving the neurovascular supply of the area, which supports recovery and minimizes potential complications [38]. For the proximal thigh soft tissue defect, commonly used muscles for myocutaneous flaps include gluteal muscles, tensor fasciae latae, biceps femoris, rectus femoris, and the vertical rectus abdominis flap. For reconstructions in the distal thigh, the vastus lateralis and gracilis muscles are frequently utilized [38]. Around the knee joint, the medial or lateral gastrocnemius muscles can be pivoted to cover defects, and for larger defects, they may be combined with the soleus muscle for additional coverage. In regions with high mechanical demands, such as the sole of the foot, a well-vascularized neurofasciocutaneous flap, such as the sural flap, is preferred (Figure 7) [39]. For extensive defects or limited local vascular supply, free flaps are necessary, whose vascular supply is re-anastomosed to nearby blood vessels to restore blood supply, allowing them to integrate well into the defect area [40]. Free flaps are preferred following oncologic excision, as the elongation of the surgical incision for a local flap may expand the extent of tumor contamination if the tumor has not been completely resected. The rectus abdominis or latissimus dorsi muscle is mostly used for this purpose [38].

**Figure 8:**
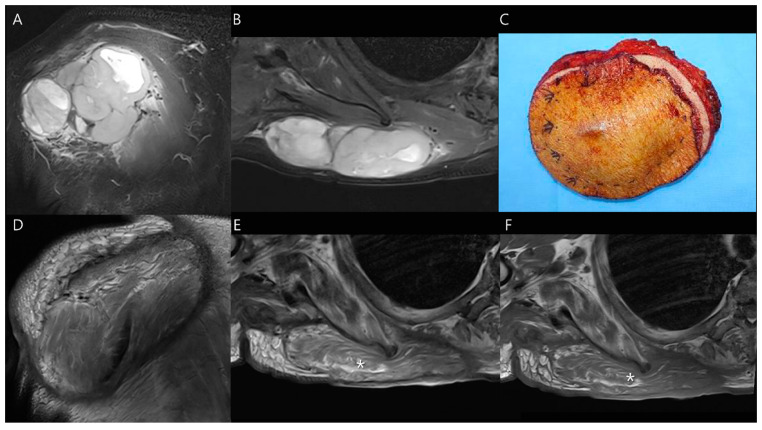
Wide excision for undifferentiated pleomorphic sarcoma on the posterior chest wall. (**A**,**B**) Preoperative coronal and axial T2-weighted fat-suppressed MR images show a 10 cm soft tissue mass. (**C**) The tumor was removed through a wide excision, and the resultant defect was covered with a free latissimus dorsi flap. (**D**,**E**) Postoperative coronal and axial T2-weighted MR images after 6 months demonstrate successful coverage of the defect by the flap, confirming proper flap integration with surrounding tissues. Note fatty infiltration within the flap area (asterisk in **E**). (**F**) Axial T1-weighted MR image shows fatty infiltration within the flap area (asterisk).

Myocutaneous flaps undergo predictable changes over time. They initially show increased edema and inflammation, which manifest as increased signal intensity on T2-weighted images. In approximately one-third of cases, this signal intensity normalizes to resemble that of adjacent healthy muscle within about 2 years. Over time, the flaps undergo atrophy, with a decrease in muscle volume and replacement by fat (Figure 8) [36]. In terms of blood flow, the flaps demonstrate increased blood flow, as evidenced by increased enhancement on contrast-enhanced MRIs. However, in about one-third of cases, this enhancement resolves within 18 months [37].

## 4. Unexpected Postoperative Imaging Findings

### 4.1. Postoperative Complication of Endoprostheses for Bone Tumors

Patients undergoing limb-salvage surgery with tumor prostheses face a higher risk of complications compared to conventional joint replacement procedures [24]. The presence of large metallic implants and extensive soft tissue removal increases the risk of early postoperative infections, wound issues, joint instability, implant dislocation (especially after a hemipelvic prosthesis), and nerve and vascular injury [41]. Long-term complications included delayed infection, aseptic loosening, and mechanical failure [42,43,44]. Delayed infections, often requiring prosthesis removal, antibiotic treatment, and potential re-implantation or amputation, are associated with worse outcomes. As treatment improves, aseptic loosening is expected to become a significant long-term complication, and mechanical failure may also occur [41]. Although clinical information, such as patient history, physical examination, and laboratory findings, can offer valuable clues for assessing infection, aseptic loosening, and mechanical failure, here, we have emphasized and provided details only on imaging approaches.

#### 4.1.1. Periprosthetic Infection

**Figure 9:**
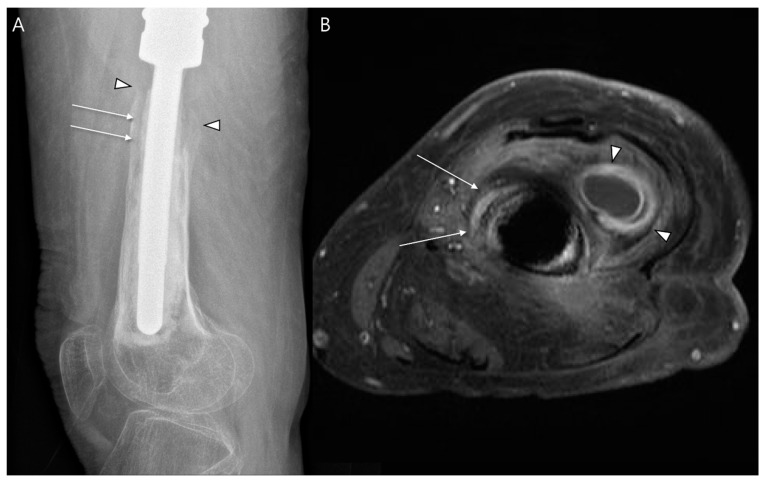
Periprosthetic infection following limb-salvage surgery for osteosarcoma performed 15 years ago. (**A**) Postoperative plain radiograph of the distal femur shows osteolysis surrounding the femoral stem of an endoprosthesis, measuring more than 2 mm (arrows), with blurred cortical remodeling (arrowheads). (**B**) Axial T1-weighted enhanced MR image shows periosteal enhancement (arrows) and soft tissue abnormalities with abscess formation (arrowheads), indicating periprosthetic infection rather than implant loosening.

Imaging findings indicative of periprosthetic infection, particularly delayed infection that occurs more than 2 years after surgery, can be challenging to differentiate from mechanical loosening. Both conditions often progress gradually and present similar imaging findings [45]. A frequent radiographic finding associated with infection is osteolysis, which appears as a lucent area at the bone–hardware interface, measuring greater than 2 mm per year. While such lucency may suggest aseptic loosening, certain features can strongly indicate a periprosthetic infection. Specifically, a rapid progression of lucency, the presence of periosteal reaction, and multifocal zones of osteolysis with indistinct edges at the margins of the prosthesis are significant markers that favor the diagnosis of infection (Figure 9) [45,46]. An MRI is particularly advantageous due to its ability to identify soft tissue abnormalities more effectively than radiographs or CT scans (Figure 9). This capability enhances the overall diagnostic accuracy and helps differentiate between infections and other complications in cases of suspected periprosthetic infections [25].

#### 4.1.2. Aseptic Loosening

**Figure 10:**
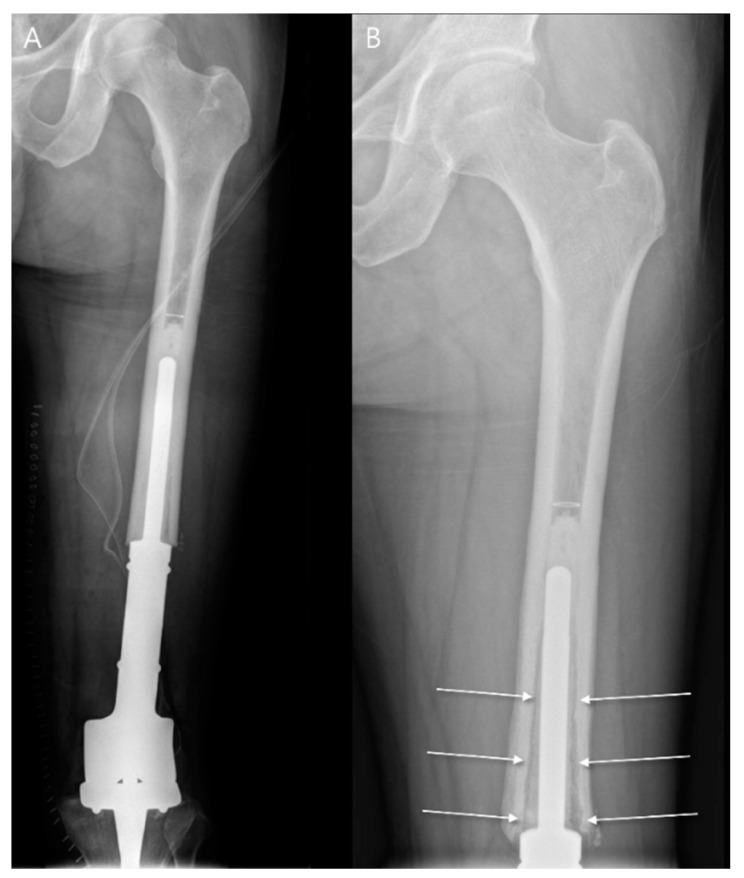
Aseptic loosening following limb salvage surgeries for low-grade central osteosarcoma. (**A**) Immediate postoperative plain radiograph is shown. (**B**) One-year follow-up plain radiograph shows a diffuse radiolucent zone at the metal–bone interface, measuring more than 1 mm (arrows), suggesting the implant is “probably loose”.

In cases where aseptic loosening is suspected, it is essential to evaluate postoperative radiographs by comparing them with the most recent available imaging. Each zone should be carefully inspected for signs of osteolysis and any radiolucent areas greater than 1 mm at the prosthesis–bone interface. Specific criteria can help classify the likelihood of loosening: “possibly loose”, if there is a radiolucent area covering between 50% and 100% of the prosthesis–bone interface; “probably loose”, if there is a continuous radiolucent line around the prosthesis, though no migration of the implant has occurred (Figure 10); “definitely loose”, if there is observable migration of the prosthesis, indicating the need for surgical revision [47].

#### 4.1.3. Mechanical Failure

**Figure 11:**
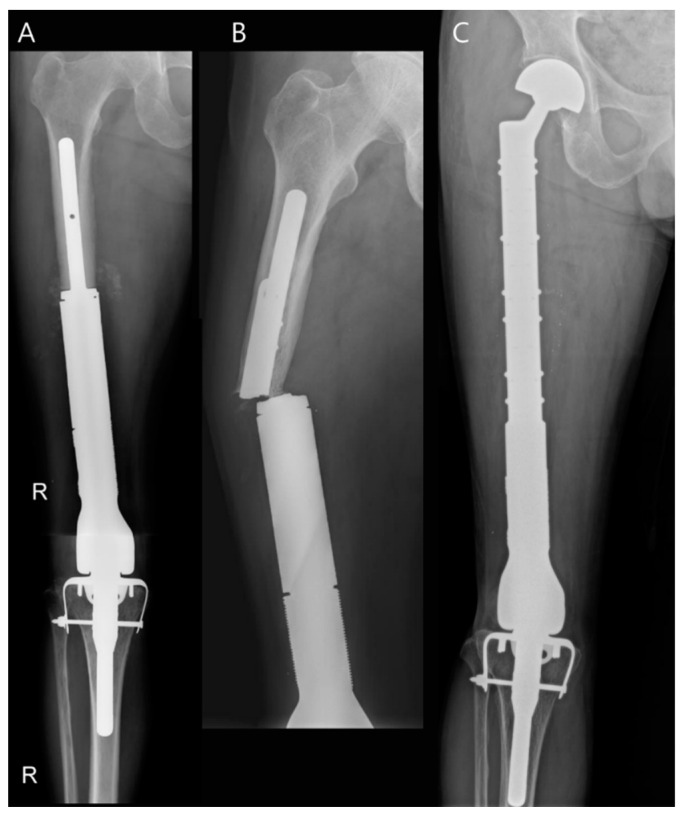
Mechanical failure following revisional limb-salvage surgery for osteosarcoma. (**A**) Immediate postoperative plain radiograph is shown (The letter R is an abbreviation for right). (**B**) Follow-up plain radiograph shows a fracture at the base of a prosthetic stem. (**C**) Plain radiograph after revisional limb-salvage surgery is shown.

Aseptic loosening is recognized as the most common cause of mechanical failure [43]. Another significant cause of mechanical failure includes fractures, which may involve either the periprosthetic area or the prosthesis itself (Figure 11). These types of fractures are classified as Type 3 failures according to the Henderson Failure Mode Classification [48]. The radiologist must evaluate for the following: widened interfaces between the prosthesis and bone (which can resemble infection), (peri)prosthetic fracture, and serial positional changes over time on radiographs [49,50].

### 4.2. Postoperative Complication of Reconstructive Surgery for Soft Tissue Tumors

Common complications after soft tissue reconstructive surgery include the formation of seroma or hematoma, infection, and denervation-induced muscle change [40,51]. The risk of postoperative complications is heightened when additional radiation therapy is administered [52]. Adjuvant therapies or additional surgeries for local recurrences can also elevate the risk of venous thromboembolism [53].

#### 4.2.1. Seroma

**Figure 12:**
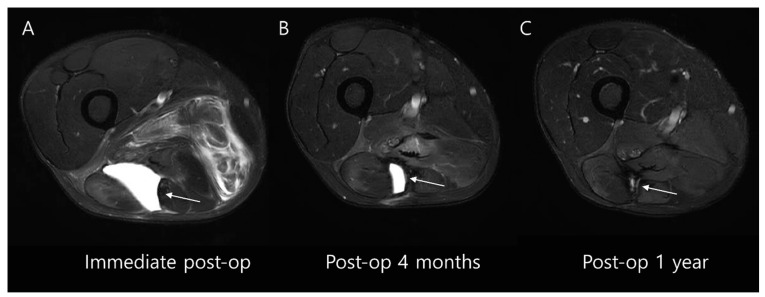
Postoperative seroma following wide excision of atypical lipomatous tumor in the thigh. (**A**) Immediate postoperative axial T2-weighted fat-suppressed MR image shows a well-defined, homogeneous fluid collection at the surgical site (arrow). (**B**) Axial T2-weighted fat-suppressed MR image taken 4 months after tumor resection demonstrates partial reduction in the size of the fluid collection (arrow). (**C**) One year post-surgery, axial T2-weighted fat-suppressed MR image shows near-complete resolution of fluid collection within the surgical site (arrow).

Seroma formation is a common complication that can arise following surgery for soft tissue sarcoma. It manifests as a well-defined, fluid-filled lesion within the surgical site. On MRIs, seromas typically appear as low or intermediate signal intensity on T1-weighted images compared to the surrounding muscle, and as very high signal intensity on T2-weighted images, indicative of the fluid content (Figure 12) [54]. Occasionally, a thin rim enhancement may be visible on contrast-enhanced MRI scans [55]. Although most seromas tend to gradually resolve within 3 to 18 months, some may persist much longer [56].

#### 4.2.2. Hematoma

**Figure 13:**
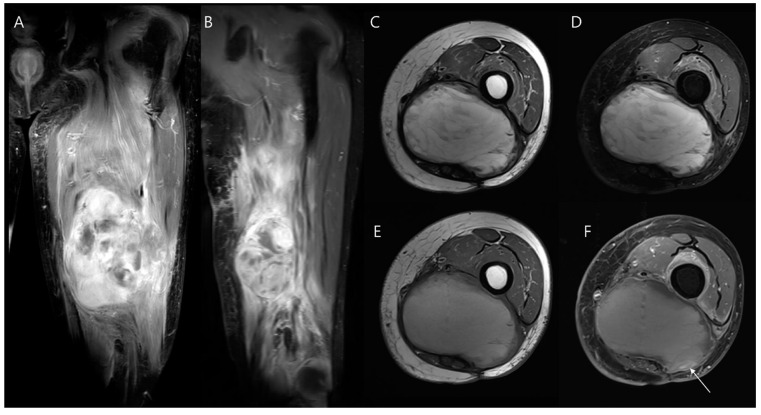
Chronic expanding hematoma after two wide excisions for myxofibrosarcoma in the thigh. (**A**) Initial coronal T1-weighted fat-suppressed enhanced MR image shows a large soft tissue mass with areas of necrosis and nodular enhancement, necessitating a wide excision. (**B**) Follow-up coronal T1-weighted fat-suppressed enhanced MR image shows a recurrence of the mass with a discrete margin and round shape, leading to a second wide excision. One year post-surgery following the final wide excision, axial (**C**) T2-weighted, (**D**) T2-weighted fat-suppressed, (**E**) T1-weighted, and (**F**) T1-weighted fat-suppressed enhanced MR images show a well-defined, hemorrhagic, mass-like lesion with peripheral nodular enhancement (arrow in (**F**)) within the surgical site. This lesion exhibited slow growth, and debulking surgery was performed to address the chronic expanding hematoma.

Postoperative hematomas often present as lobulated, mass-like formations with both solid and cystic components, depending on their stage of maturation. These lesions are typically inhomogeneous in appearance due to the presence of different blood products at varying stages of degradation [40]. Using fat suppression and subtraction imaging techniques can help differentiate a hematoma from a recurrent mass, enhancing diagnostic accuracy [36]. However, chronic expanding hematomas can make evaluation of the surgical site challenging [57]. These hematomas grow slowly and often show nodular enhancement, likely due to repeated capillary injury and bleeding caused by ongoing irritation from blood products (Figure 13) [58,59,60]. In such cases, definitive exclusion of tumor recurrence typically requires a biopsy to confirm the diagnosis and exclude recurrence [58,61].

#### 4.2.3. Wound Infection

**Figure 14:**
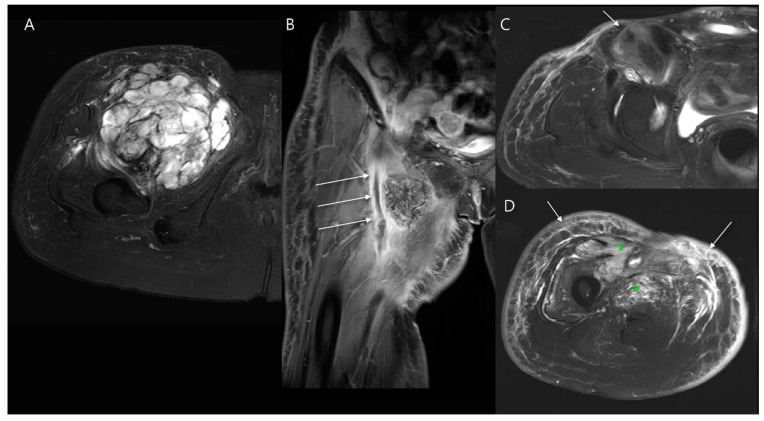
Wound infection. (**A**) Preoperative axial T2-weighted fat-suppressed MR image shows a myxofibrosarcoma in the proximal thigh. Neoadjuvant chemotherapy was administered, followed by wide excision, femoral nerve neurolysis, and a femoral-femoral bypass procedure. (**B**) Postoperative coronal T1-weighted fat-suppressed enhanced MR image shows an internal thrombus within the bypass (arrows). (**C**,**D**) Axial T2-weighted fat-suppressed images demonstrate herniation of the small bowel through the large defect (arrow in (**C**)), along with cellulitis (arrows in (**D**)) and myositis (asterisks in (**D**)).

Patients with diabetes, prolonged surgical procedures, large incisions, extensive soft tissue defects, perioperative radiotherapy, and immune deficiency due to perioperative chemotherapy are more susceptible to infection [62,63]. High signal intensity soft tissue abnormalities, visible on T2-weighted MR images, may indicate the presence of an infection (Figure 14) and can be associated with abscess formation [55].

#### 4.2.4. Denervation-Induced Muscle Change

**Figure 15:**
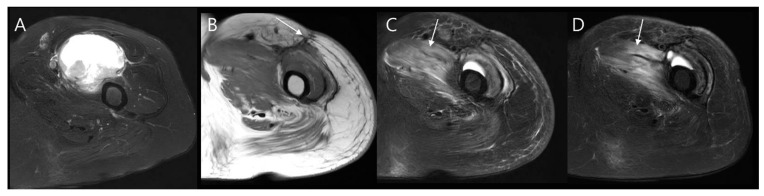
Denervation-related muscle changes. (**A**) Preoperative axial T2-weighted fat-suppressed MR image shows a synovial sarcoma in the proximal thigh. A wide excision with femoral vessel ligation was performed. (**B**) Postoperative axial T1-weighted image taken 7 months later reveals a fibrotic scar at the incisional site (arrow). (**C**) Axial T2-weighted, fat-suppressed image shows edematous change due to denervation in areas innervated by the femoral nerve (arrow). (**D**) Axial T2-weighted, fat-suppressed image taken 1 year postoperatively shows partial regression of the edema, along with volume loss in the affected muscle (arrow).

During musculoskeletal tumor surgeries and related procedures, there is a risk of nerve entrapment or transection, which can lead to changes in the muscle tissue due to denervation [37,51,64]. MRIs can detect these nerve injuries by showing areas of increased signal intensity on T2-weighted images in the affected muscles. This denervation-related muscle edema may evolve over time, and within 6–12 months after the initial injury, these acute changes can progress to chronic atrophic change, characterized by muscle volume loss and fatty infiltration (Figure 15) [51].

### 4.3. Postoperative Complication After Amputation

Amputation neuroma is an uncommon complication that can arise following limb amputation, characterized by localized swelling or thickening of nerve tissue just proximal to the surgical stump [65]. This condition results from hyperplasia of the neuronal fascicles, which originally led to it being referred to as “paradoxical diffuse hypertrophy”. This hyperplasia is marked by disorganized nerve fibers and the formation of perineural fibrous tissue around the nerve [66].

**Figure 16:**
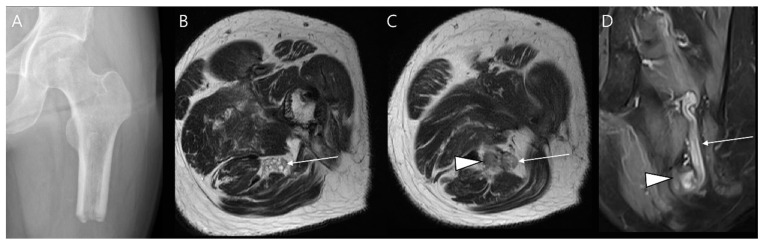
Amputation neuroma. (**A**) A 48-year-old man with a history of osteosarcoma who underwent an above-the-knee amputation. (**B**,**C**) Axial T2-weighted MR images obtained 1 year post-surgery reveal enlargement of the fascicles along the sciatic nerve (arrows). A bulbous mass is visible at the end of the sciatic nerve (arrowhead), suggesting an amputation neuroma. (**D**) Coronal T1-weighted fat-suppressed contrast-enhanced MR image shows an ovoid mass with minimal enhancement (arrowhead) at the end of the sciatic nerve (arrow).

On imaging studies, amputation neuroma typically appears as a bulbous mass on or fusiform thickening along the nerve, while the nerve itself remains continuous proximally (Figure 16). This continuity is a critical feature for distinguishing amputation neuromas from tumor recurrence at the stump. Amputation neuromas are generally painless, exhibit diffuse thickening, and do not show contrast enhancement in imaging studies [65,67,68].

The summary of postoperative complications is presented in Table 1.

## 5. Local Recurrence

### 5.1. Guidelines for Imaging Surveillance

One of the most significant concerns in managing oncologic surgeries is the risk of tumor recurrence [48,69,70,71,72]. While specific follow-up protocols can vary based on tumor characteristics, general guidelines for imaging surveillance have been established.

- According to the American College of Radiology (ACR) Appropriateness Criteria, imaging should be performed every 3–6 months during the first 10 years, with annular MRI scans recommended between Years 5 and 10, along with any additional imaging based on new symptoms [73].
- The European Society of Skeletal Radiology (ESSR) recommends imaging every 3–4 months during the first 3 years, every 6 months from Years 3 to 5, and then annual follow-ups from Years 5 and 10 [74].

For malignant bone tumors, especially when assessing local recurrence, both radiography and MRIs are used to monitor the surgical site and surrounding tissues [73]. Interval MRIs have proven particularly effective in detecting recurrent tumors within the surgical bed, providing critical information for timely intervention [75,76,77,78].

The likelihood of local recurrence is influenced by various factors, including tumor biology, patient demographics, and treatment approaches [79,80,81,82,83]. A study from M.D. Anderson Cancer Center, involving 1225 patients with localized primary soft tissue sarcomas, identified several factors significantly associated with local recurrence [84]:

- Tumor biology: (i) aggressive histological subtypes, such as undifferentiated pleomorphic sarcoma; (ii) high-grade tumors; (iii) large tumor size (>10 cm) in its greatest dimension
- Patient demographics: older age (>64 years)
- Treatment factors: (i) positive or uncertain resection margins; (ii) previous recurrence
- Tumor location: head, neck, or deep trunk

### 5.2. Imaging Appearance of Local Recurrence

MRIs employ various sequences that, when interpreted collectively, allow radiologists to differentiate between local tumor recurrence and typical postoperative complications, such as seromas, hematoma, inflammation, and scar tissue formation [75,79]. Intravenous contrast administration can enhance the visibility of recurrent tumors, making them easier to detect [75,85]. Standard MRI protocols typically include T1-weighted image (T1WI), T2-weighted image (T2WI), T2WI with fat suppression (FS), and T1WI FS with contrast enhancement. Axial and coronal or sagittal views are commonly used, with adjustments to the field of view (FOV) to optimize image resolution or anatomical coverage. Advanced imaging techniques, such as diffusion-weighted imaging (DWI) and dynamic contrast enhancement (DCE)-MRI may be employed for further evaluation [86].

Imaging of tumors located in distal extremities (e.g., fingers, toes), shoulder, and pelvic girdles can be challenging. For tumors in the distal extremities, small size, complex anatomy, and motion artifacts often hinder imaging, necessitating high-resolution 3T MRI [87]. Tumors in the shoulder and pelvic girdles are frequently obscured by overlapping internal organs, such as the lung or gastrointestinal tract. Additionally, respiratory motion and bowel movements can introduce artifacts into the images, requiring advanced imaging processing techniques [88].

**Figure 17:**
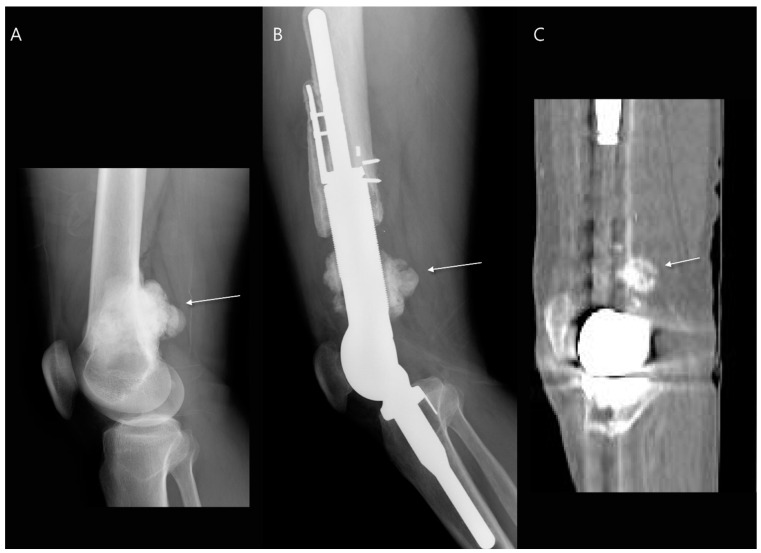
Local recurrence after limb-salvage surgery for parosteal osteosarcoma. (**A**) Preoperative radiograph reveals juxtacortical mineralization arising from the posterior aspect of the distal femur (arrow), consistent with a diagnosis of parosteal osteosarcoma. (**B**,**C**) One year later, both the radiograph and CT scan demonstrate mineralization surrounding the femoral component (arrows), indicative of recurrent osteosarcoma.

**Figure 18:**
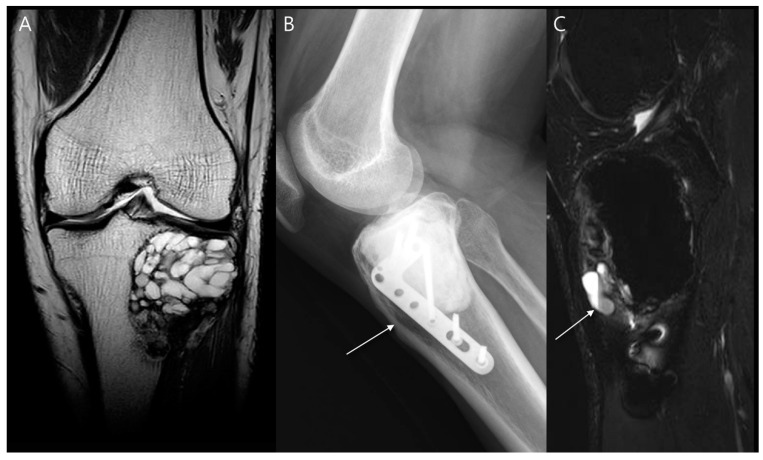
Local recurrence after intralesional curettage with cementation for giant cell tumor of bone. (**A**) Preoperative coronal T2-weighted MR image shows a well-defined osseous lesion with aneurysmal bone cyst changes in the proximal tibia. (**B**) One year later, a radiograph shows deep periprosthetic lucency anterior to the cement insertion (arrow), raising suspicion of recurrence. (**C**) Sagittal T2-weighted fat-suppressed MR image confirms the presence of a recurrent lesion with aneurysmal bone cyst changes (arrow).

Local recurrences most commonly occur at or near the site of the original tumor (Figure 17), with rare instances of recurrence manifesting at more distant sites, such as along the surgical-access pathway [89,90,91]. The signal characteristics of recurrent tumors are usually consistent with those seen in the primary tumor (Figure 18), which can help guide radiologists in identifying disease recurrence [89]. Recurrent soft tissue sarcoma typically presents on imaging as a distinct, rounded or ovoid nodule or mass (Figure 13A,B) [92,93]. Comparing current images with preoperative scans helps identify any new growth or changes in the tumor bed [92].

### 5.3. Special Consideration for Imaging Assessment of Local Recurrence

**Figure 19:**
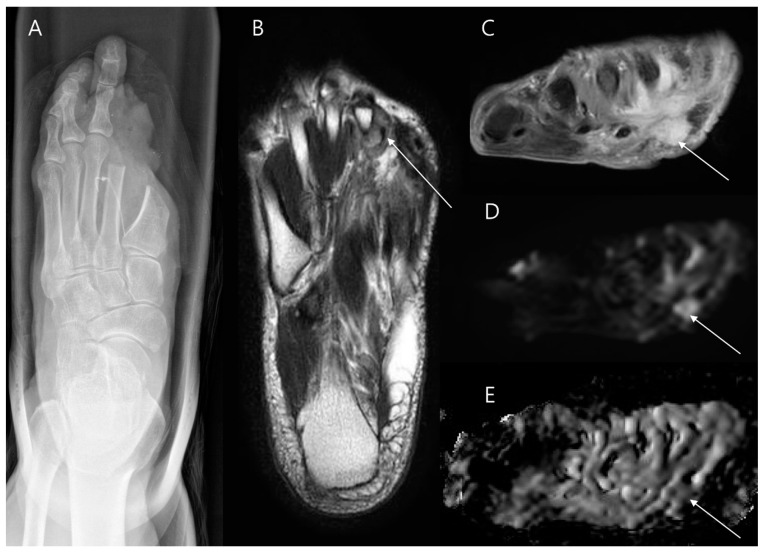
Recurrent mass in a patient with clear cell sarcoma after amputation with flap coverage. (**A**) Post-amputation state is shown. (**B**) Postoperative 3-month coronal T2-weighted MR image shows a nodular lesion near the flap (arrow). (**C**) Axial T1-weighted fat-suppressed enhanced MR image highlights a discrete enhancing nodule near the flap (arrow). (**D**,**E**) This lesion (arrows) demonstrates high signal intensity on the high b-value image of diffusion-weighted imaging and low signal intensity on the apparent diffusion coefficient map, indicating a recurrent mass.

While enhancement on imaging can be indicative of tumor recurrence, it is important to note that it is not a definitive marker. Various other factors, such as post-surgical inflammation, granulation tissue, and even rare radiation-induced pseudomasses, can also lead to similar imaging findings [52]. Dynamic contrast-enhanced MRI can assess the rate and pattern of enhancement. A steep increase in signal intensity over time on a time-intensity curve is characteristic of recurrent tumors with a true mass effect [94,95,96,97,98]. DWI is another useful technique for differentiating recurrent tumors from post-surgical scarring. DWI can detect changes in water diffusion within tissues, which can help distinguish between highly cellular recurrent tumor cells and the lower cellularity of benign scar tissue (Figure 19) [98,99,100,101].

**Figure 20:**
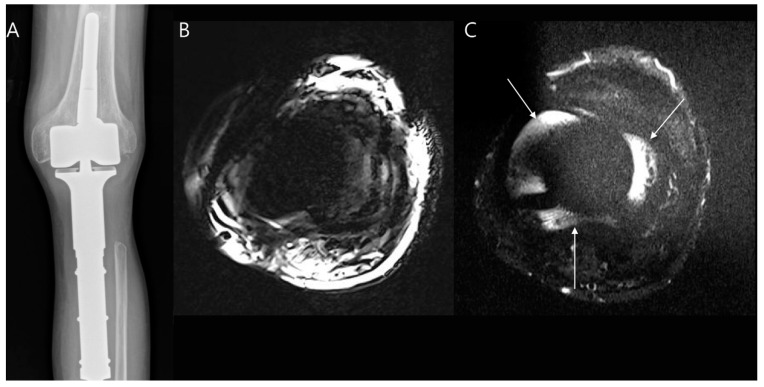
Periprosthetic fluid collection in a patient with a malignancy in giant cell tumor after limb-salvage surgery. (**A**) Limb-salvage surgery is shown. (**B**) Axial T2 Dixon water-only image is distorted by susceptibility artifact, making it difficult to visualize the periprosthetic region. (**C**) Axial T2 STIR (short-tau inversion recovery) image using the SEMAC (slice encoding for metal artifact correction) technique clearly demonstrates periprosthetic fluid collection (arrows) surrounding the tibial component.

Limb-salvage surgery for bone tumors often involves endoprostheses. Postoperative imaging is challenging due to metal artifacts caused by implants, which create susceptibility artifacts that hinder periprosthetic bone assessment [89]. Techniques like MARS (metal artifact reduction sequence), SEMAC (slice encoding for metal artifact correction, Figure 20), and VAT (view angle tilting) help reduce these artifacts, especially with 3 Tesla MRI [102,103,104,105,106]. In many cases, 1.5 Tesla scanners with specific sequences such as STIR (short-tau inversion recovery), Dixon sequences, or adjusted parameters (e.g., higher frequency coding bandwidth, thinner slices, shorter echo train length) are advised [89,107,108,109].

## 6. Conclusions

Evaluating postoperative imaging for musculoskeletal tumors requires an understanding of various surgical concepts and procedures to help identify potential complications early. For optimal postoperative monitoring, MRIs with intravenous contrast should ideally be conducted. Plain radiographs and CT scans play a crucial role, especially in cases involving primary bone tumors. Advanced imaging techniques, such as DWI and DCE-MRI, have significantly improved diagnostic accuracy in detecting tumor recurrence. Simultaneously, advancements in robotic, augmented reality, and 3D printing technologies have enhanced surgical precision and efficiency in orthopaedic oncology [110]. By tailoring imaging surveillance to the specific surgical procedure and recognizing potential complications and local recurrence, radiologists and clinicians can facilitate early diagnosis and intervention, leading to improved patient outcomes. While this framework for postoperative imaging is comprehensive, it is important to acknowledge its limitations. Future research should focus on developing advanced imaging protocols to further enhance image quality and diagnostic accuracy.

## Figures and Tables

**Table 1:** Postoperative complications in musculoskeletal tumors.

After Endoprosthesis for Bone Tumor	After Reconstructive Surgery for Soft Tissue Tumor	After Amputation
Periprosthetic infection	Seroma	Amputation neuroma
Aseptic loosening	Hematoma	
Mechanical failure	Wound infection	
	Denervation-induced muscle change	

## Data Availability

Dataset available on request from the authors.
